# Neuron-Glial Antigen 2 Participates in Liver Fibrosis via Regulating the Differentiation of Bone Marrow Mesenchymal Stem Cell to Myofibroblast

**DOI:** 10.3390/ijms24021177

**Published:** 2023-01-07

**Authors:** Le Yang, Hang Zhang, Chengbin Dong, Wenhui Yue, Renmin Xue, Fuquan Liu, Lin Yang, Liying Li

**Affiliations:** 1 Department of Cell Biology, Municipal Laboratory for Liver Protection and Regulation of Regeneration, Capital Medical University, Beijing 100069, China; 2 Department of Interventional Therapy, Beijing Shijitan Hospital, Capital Medical University, Beijing 100069, China

**Keywords:** liver fibrogenesis, smooth muscle actin, siRNA, TGFβ1, chimera mouse

## Abstract

Neuron-glial antigen 2 (NG2, gene name: *Cspg4*) has been characterized as an important factor in many diseases. However, the pathophysiological relevance of NG2 in liver disease specifically regarding bone marrow mesenchymal stem cell (BMSC) differentiation to myofibroblast (MF) and the molecular details remain unknown. Human liver tissues were obtained from patients with different chronic liver diseases, and mouse liver injury models were induced by feeding a methionine-choline-deficient and high-fat diet, carbon tetrachloride administration, or bile duct ligation operation. NG2 expression was increased in human and mouse fibrotic liver and positively correlated with MF markers α-smooth muscle actin (αSMA) and other fibrotic markers in the liver. There was a co-localization between NG2 and αSMA, NG2 and EGFP (BMSC-derived MF) in the fibrotic liver determined by immunofluorescence analysis. In vitro, TGFβ1-treated BMSC showed a progressive increase in NG2 levels, which were mainly expressed on the membrane surface. Interestingly, there was a translocation of NG2 from the cell membrane into cytoplasm after the transfection of *Cspg4* siRNA in TGFβ1-treated BMSC. siRNA-mediated inhibition of *Cspg4* abrogated the TGFβ1-induced BMSC differentiation to MF. Importantly, inhibition of NG2 in vivo significantly attenuated the extent of liver fibrosis in methionine-choline-deficient and high fat (MCDHF) mice, as demonstrated by the decreased mRNA expression of fibrotic parameters, collagen deposition, serum transaminase levels, liver steatosis and inflammation after the administration of *Cspg4* siRNA in MCDHF mice. We identify the positive regulation of NG2 in BMSC differentiation to MF during liver fibrosis, which may provide a promising target for the treatment of liver disease.

## 1. Introduction

Liver fibrosis is a dynamic, highly integrated molecular, cellular and tissue process of chronic liver diseases irrespective of etiology, which eventually leads to cirrhosis and related complications, including hepatocellular carcinoma [1,2]. Hepatic myofibroblast (MF), a heterogeneous population, is thought to be responsible for driving the excess accumulation of extracellular matrix components in the pathogenesis of liver fibrosis [3]; thus, elucidating the mechanisms of MF activation seems indispensable for designing rational therapeutic strategies to inhibit the fibrogenic process leading to cirrhosis. It is well documented that a high proportion of MF is of bone-marrow origin in the fibrotic liver [4,5]. For instance, our previous studies have demonstrated that bone marrow mesenchymal stem cells (BMSC) can migrate to the damaged liver and differentiate to α-smooth muscle actin (protein name: αSMA, mouse gene name: *Acta2*, human gene name: *ACTA2*)-positive MF in the fibrotic liver [6,7,8]. Therefore, targeting the critical regulation factors in the differentiation of BMSC to MF in the development of hepatic fibrosis is viewed as a promising strategy for the treatment of liver disease.

Neuron-glial antigen 2 (protein name: NG2, mouse gene name: *Cspg4*, human gene name: *CSPG4*), also known as chondroitin sulfate proteoglycan 4 (CSPG4) or melanoma-associated chondroitin sulfate proteoglycan, is a membrane-spanning proteoglycan, consisting of an N-linked 280 kD glycoprotein component and a 450 kD chondroitin sulfate proteoglycan component expressed on the membrane of cells [9,10]. The 280 kD and 450 kD components of NG2 contain the same core protein [11]. NG2 protein was originally detected by antibodies in a cell line of rats with glial and neuronal properties where it is thought to play a role in regulating the blood-brain barrier and tissue homeostasis [12,13,14]. Thus, the initial research regarding NG2 was focused on its role in the central nervous system, finding that NG2^+^ glia cells (glia cells expressing NG2) represent the oligodendrocyte precursor cells, the most abundant population of endogenous/resident progenitor cells, which can react to injury and potentially repopulate areas of the lesion and regulate axon regeneration. For instance, genetic mutation of *CSPG4* led to the dysfunction of oligodendrocyte progenitor cells and failure to differentiate into myelinating oligodendrocytes in familial schizophrenia [15]. Recent studies have identified that NG2 functions in various cell types and NG2 levels are increased in many aggressive cancers, including melanomas [16], gliomas [17], mesotheliomas [18], sarcomas [19], anaplastic thyroid cancer [20], triple-negative breast cancers [21] and hepatocellular carcinoma [22]. Moreover, NG2 overexpression may induce highly aggressive tumors characterized by increased angiogenesis and a moderately invasive phenotype [23,24]. However, the pathophysiological relevance of NG2 in liver disease specifically regarding MF activation and the molecular details have not yet been determined.

In the present study, we analyzed NG2 expression in the fibrotic livers of patients and multiple mouse models and identified the critical role of NG2 in the differentiation of BMSC to MF during liver fibrosis. Importantly, in vivo administration of *Cspg4* siRNA significantly attenuates the extent of liver fibrogenesis. These results represent the first experimental evidence that NG2 is involved in BMSC differentiation to MF during liver fibrosis, which may open new perspectives for the pharmacological treatment of liver disease.

## 2. Results

### 2.1. The Dynamic Changes in NG2 Expression in the Fibrotic Livers of Patients and Multiple Mouse Models

**Figure 1:**
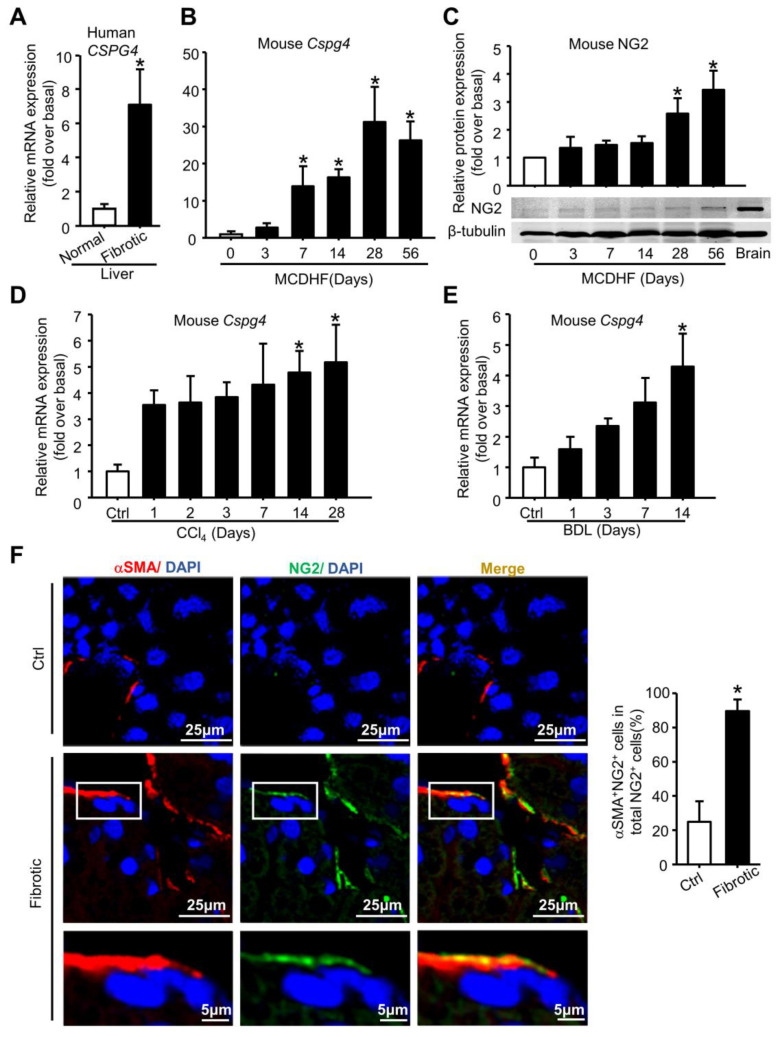
The dynamic changes in hepatic NG2 levels in fibrotic liver of patients and multiple mouse models. (**A**) *CSPG4* mRNA expression was examined in the fibrotic liver of patients. *n* = 28. (**B**) *Cspg4* mRNA expression was examined by qRT-PCR in the fibrotic liver induced by feeding an MCDHF diet. *n* = 3 per group. (**C**) NG2 protein expression was examined by Western blot in the fibrotic livers of MCDHF mice. *n* = 3 per group. (**D**) *Cspg4* mRNA expression was examined in the fibrotic liver induced by CCl_4_ administration. *n* = 4 for CCl_4_ 0 day, 5 for CCl_4_ 3 days, 6 for CCl_4_ 1, 2, 7, 14 days and 28 days control per group, 9 for 28 days (**E**) *Cspg4* mRNA expression was examined in the fibrotic liver of BDL mouse models of liver fibrosis. *n* = 3 for BDL 0, 3 days per group, 4 for BDL 1, 14 days per group, and 5 for BDL 7 days. (**F**) Immunofluorescent staining for NG2 (green) and αSMA (red) to track their expression in the fibrotic liver following 28 days of an MCDHF diet. Pictures in the third row are taken from the fields that are indicated as “white box” in the second row. DAPI (blue) was used to visualize nuclei. The proportion of NG2^+^αSMA^+^ cells accounting for total NG2^+^ cells was measured by Image-Pro Plus software (version 5.0). Data are presented as the mean ± SEM. * *p* < 0.05 vs. control.

To investigate the role of NG2 in liver fibrosis, we first examined the expression of *CSPG4* in patient fibrotic livers of different etiologies and observed a significant increase in *CSPG4* expression (Figure 1A). Further, we examined the expression of NG2 in the fibrotic liver of multiple mouse models. The hepatic mRNA levels of *Cspg4* were drastically increased from 7 days after feeding a methionine-choline-deficient and high fat (MCDHF) diet and continued to increase in a way correlated with the progression of liver fibrosis, with a maximal increase (31.24 fold) at 28 days after feeding an MCDHF diet (Figure 1B). Correspondingly, the protein level of NG2 was also increased at the indicated time after feeding an MCDHF diet, with its expression in brain tissues as positive control (Figure 1C). A similar increase in *Cspg4* mRNA levels was obtained in the fibrotic liver of carbon tetrachloride (CCl_4_) (Figure 1D) and bile duct ligation (BDL) (Figure 1E) mice. To track NG2 expression in the fibrotic liver, immunofluorescence staining was performed in MCDHF mice with demonstrated fibrosis [25] showing that NG2 was strongly expressed in the fibrotic liver, with a co-localization with MF typical marker αSMA. A large proportion of NG2^+^ cells (cells expressing NG2) were positive for αSMA, with the percentage of NG2^+^αSMA^+^ cells accounting for 89.6% of total NG2^+^ cells (Figure 1F). Above all, these results indicated the universal and critical role of NG2 in patients and multiple liver fibrotic models of mice.

### 2.2. The Correlation between CSPG4 and Fibrosis Markers in Human and Mouse Liver

**Figure 2:**
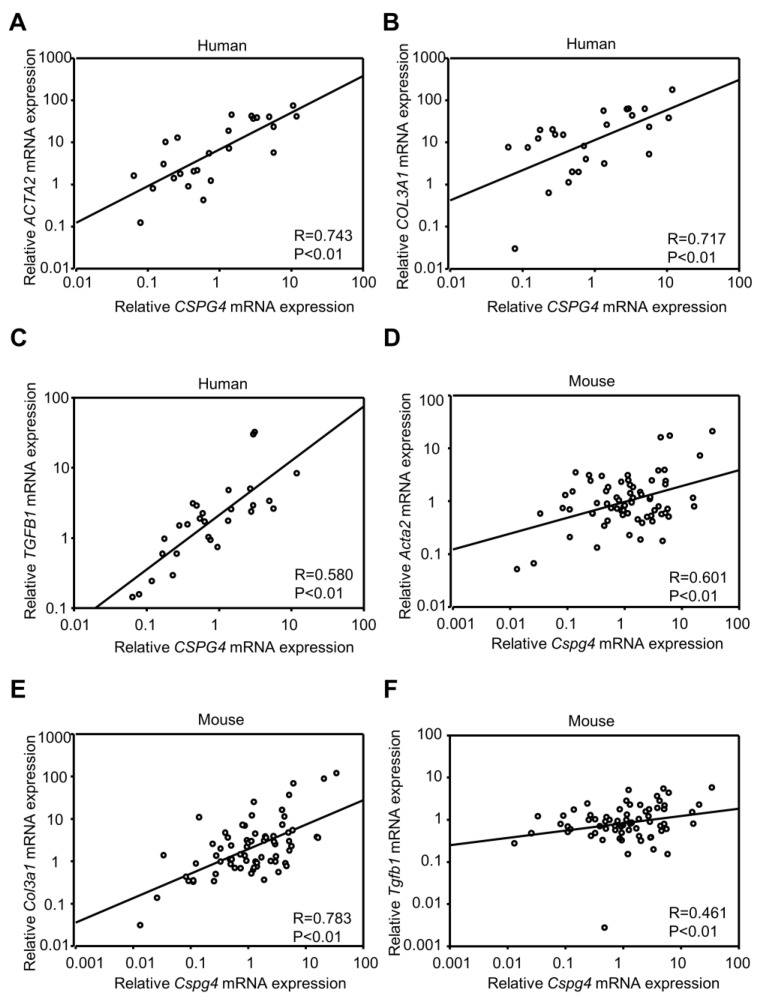
The positive correlation between *CSPG4* and fibrosis markers in human and mouse liver. The correlation between the hepatic mRNA levels of *Cspg4* and fibrotic parameters, including *Acta2* (**A**), *Col3a1* (**B**) and *Tgfb1* (**C**) in mouse liver. *n* = 69. The correlation between the hepatic mRNA levels of *CSPG4* and *ACTA2* (**D**), *COL3A1* (**E**) and *TGFB1* (**F**) in human liver. *n* = 28.

Next, we analyzed the correlation between the mRNA expression of *CSPG4* and fibrosis markers in human and mouse livers using linear correlation analysis (Figure 2). Our results showed that *CSPG4* mRNA level was positively correlated with the expression of MF marker *ACTA2* in patient livers of different etiologies (Figure 2A, r = 0.743). Moreover, we examined the correlation between the expression of *Cspg4* and *Acta2* in mouse liver, and also obtained a positive correlation (Figure 2D, r = 0.601). We further analyzed the correlation between the hepatic expression of *CSPG4* and other fibrotic parameters, including procollagen α1(III) (mouse gene name: *Col3a1*, human gene name: *COL3A1*) and transforming growth factor β1 (protein name: TGFβ1, mouse gene name: *Tgfb1*, human gene name: *TGFB1*), showing a positive correlation between them in both human and mouse liver. These results suggested that NG2 might be involved in MF activation in both human and mouse liver fibrosis, independent of species and etiology.

### 2.3. The High Expression of NG2 on BMSC-Derived MF in Fibrotic Liver

**Figure 3:**
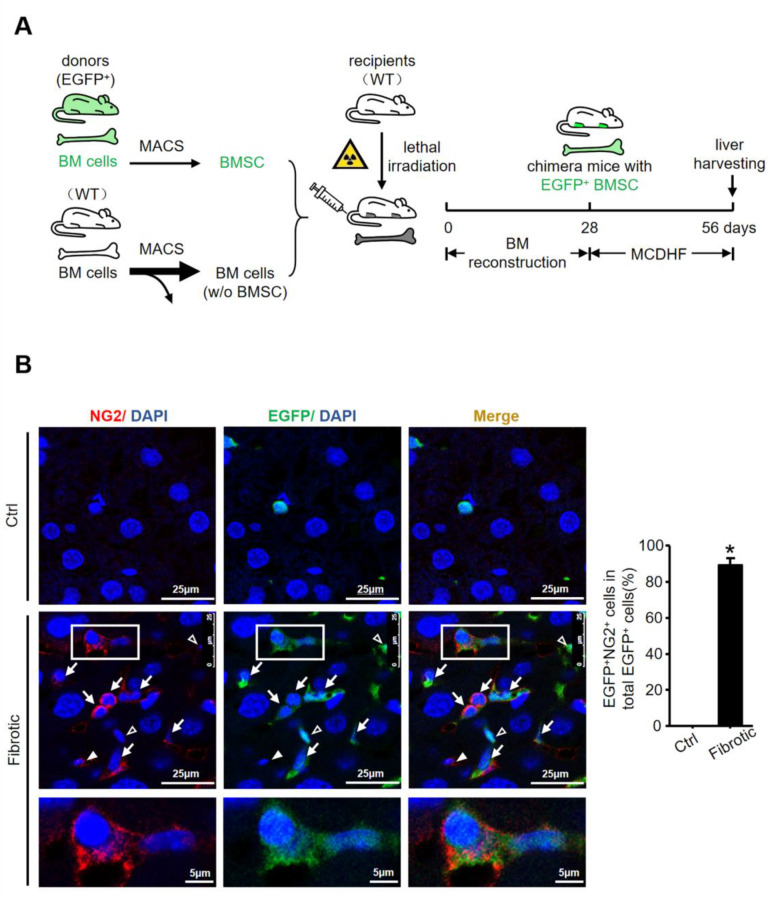
The high expression of NG2 on BMSC-derived MF in fibrotic liver. (**A**) Schematic images of the strategy for generating chimera mice with EGFP^+^ BMSC, followed by MCDHF-induced liver fibrosis. (**B**) Representative images of NG2 (red), EGFP (green) and merged fields from immunofluorescence analysis to visualize NG2 expression in BMSC-derived MF in the fibrotic liver. Pictures in the third row are taken from the fields that are indicated as “white box” in the second row. Hollow arrows indicate NG2^−^EGFP^+^ cells (cells expressing EGFP but not NG2), solid arrows indicate NG2^+^EGFP^−^ cells (cells expressing NG2 but not EGFP), and arrows indicate NG2^+^EGFP^+^ cells. DAPI (blue) was used to visualize nuclei. The proportion of NG2^+^EGFP^+^ cells accounting for total EGFP^+^ cells was measured by Image-Pro Plus software. Data are presented as the mean ± SEM. * *p* < 0.05 vs. control.

To further identify the source of NG2-expressed MF, NG2 expression was then examined in the fibrotic liver of chimera mice with EGFP^+^ BMSC by immunofluorescence staining. A schematic overview showing the strategy for generating chimera mice with EGFP^+^ BMSC using anti-CD146 microbeads, followed by feeding an MCDHF diet to induce liver fibrosis is given in Figure 3A. A co-localization was observed between NG2^+^ and EGFP^+^ cells in the fibrotic liver (Figure 3B). A large proportion of EGFP^+^ cells were positive for NG2, with the percentage of NG2^+^EGFP^+^ cells accounting for 89.2% of total EGFP^+^ cells. Previously we found that approximately 70% of the BMSC homing to the hepatic fibrosis region differentiated into MF and expressed αSMA [7]. Therefore, these results indicated that NG2 was highly expressed on these MF, which were derived from BMSC in the fibrotic liver, and probably involved in the differentiation of BMSC to MF during liver fibrosis.

### 2.4. The NG2 Expression in TGFβ1-Induced BMSC Differentiation to MF

**Figure 4:**
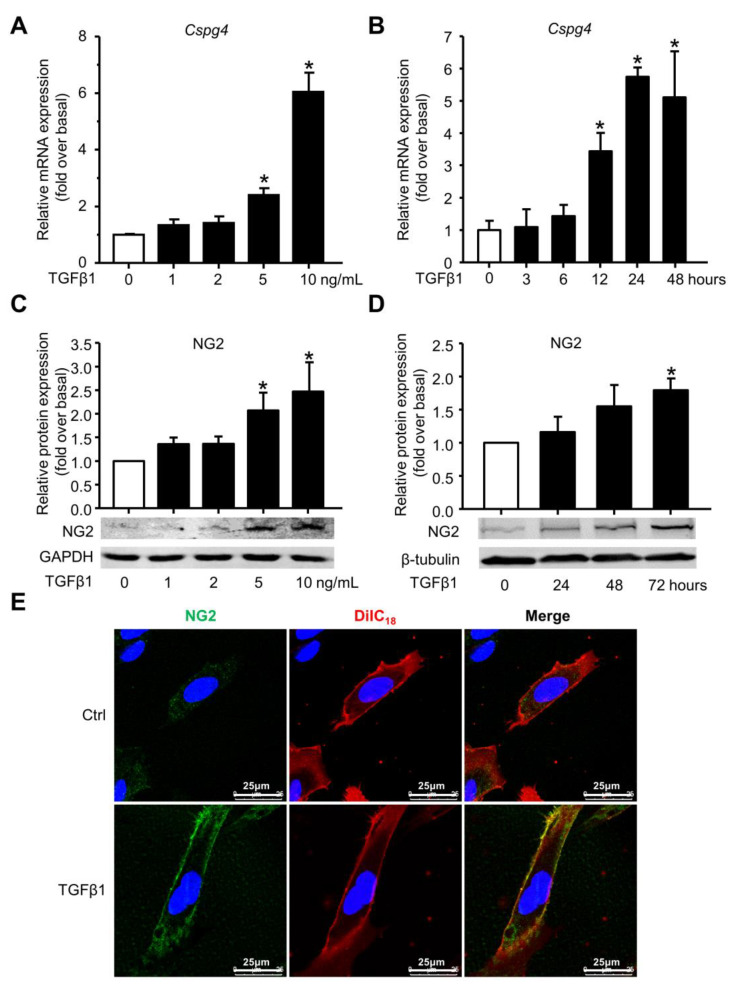
The mRNA and protein expression of NG2 in TGFβ1-induced BMSC differentiation. (**A**) *Cspg4* mRNA expression in BMSC treated with the indicated concentrations of TGFβ1 for 24 h. (**B**) *Cspg4* mRNA expression in BMSC treated with 10 ng/mL TGFβ1 for different time points. (**C**) NG2 protein expression in BMSC treated with the indicated concentrations of TGFβ1 for 24 h. (**D**) NG2 protein expression in BMSC treated with 10 ng/mL TGFβ1 for different time points. (**E**) Immunofluorescent staining for NG2 (green) in TGFβ1-treated BMSC. Cell membrane was labeled with a membrane dye DiIC_18_ (red). DAPI was used to visualize nuclei (blue). Data are presented as the mean ± SEM. *n* = 3 per group. * *p* < 0.05 vs. control.

To validate the expression of NG2 in vitro, we treated primary BMSC with TGFβ1, which is an important regulator of BMSC differentiation to MF during liver fibrosis [25]. qRT-PCR analysis revealed a pronounced increase in *Cspg4* mRNA expression stimulated by TGFβ1 in a dose-dependent manner, with the maximum up-regulation at a concentration of 10 ng/mL TGFβ1 (Figure 4A). Then BMSC were stimulated by 10 ng/mL TGFβ1 for different times, showing a progressive increase in *Cspg4* mRNA levels from 3 to 24 h, with the highest expression at 24 h (Figure 4B). NG2 protein levels were examined by Western blot. TGFβ1 treatment markedly up-regulated NG2 protein levels, with the maximum at a concentration of 10 ng/mL TGFβ1 (Figure 4C), and these elevated levels of NG2 persisted for at least 72 h (Figure 4D). We also performed immunofluorescent staining to show NG2 expression and the morphological changes in BMSC under TGFβ1 treatment. DiIC_18_, a membrane dye, was used to label the cell membrane in BMSC. Under normal conditions, isolated BMSC showed very weak expression of NG2, while treatment of TGFβ1 in BMSC markedly enhanced the protein expression of NG2, which was mainly expressed on the membrane surface (Figure 4E). Altogether, these results well demonstrated the remarkable increase in NG2 mRNA and protein levels under the treatment of TGFβ1 in the differentiation of BMSC to MF.

### 2.5. The Involvement of NG2 in TGFβ1-Induced BMSC Differentiation to MF

**Figure 5:**
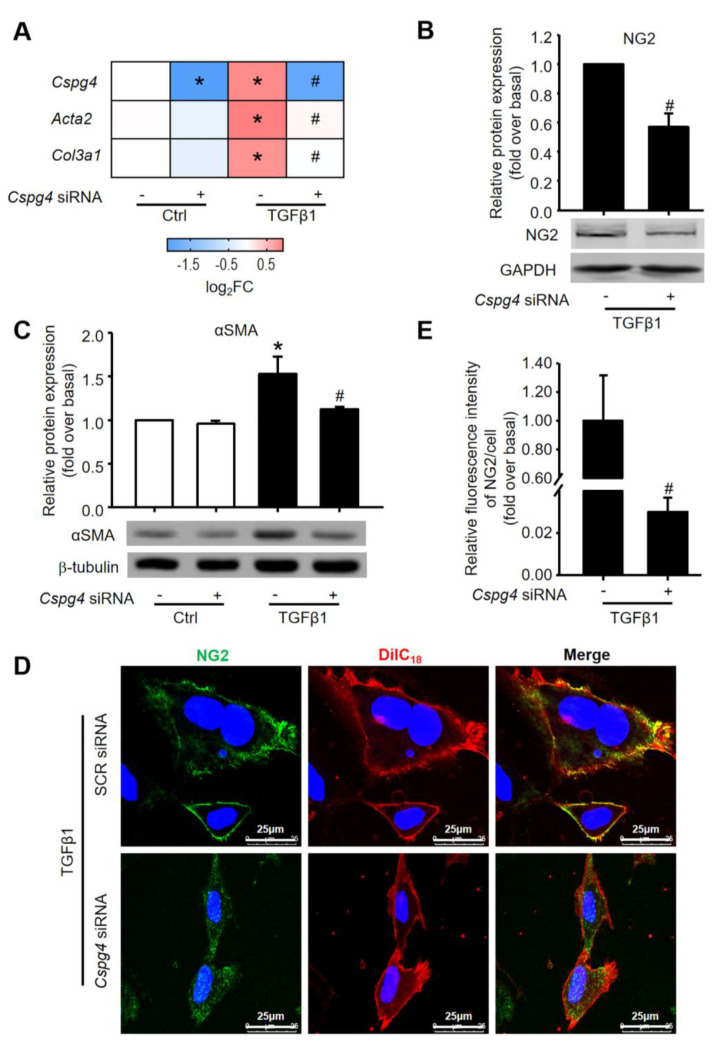
The involvement of NG2 in TGFβ1-induced BMSC differentiation. BMSC were transfected with *Cspg4* siRNA and followed by 24 h of TGFβ1 treatment. (**A**) The mRNA expression of *Cspg4*, *Acta2* and *Col3a1* in TGFβ1-treated BMSC in the presence or absence of *Cspg4* siRNA. (**B**) The protein expression of NG2 in TGFβ1-treated BMSC in the presence or absence of *Cspg4* siRNA. (**C**) The protein expression of αSMA in TGFβ1-treated BMSC in the presence or absence of *Cspg4* siRNA. (**D**) Immunofluorescent staining for NG2 (green) in TGFβ1-treated BMSC in the presence or absence of *Cspg4* siRNA. Cell membrane was labeled with a membrane dye DiIC_18_ (red). DAPI was used to visualize nuclei (blue). (**E**) Quantitative analysis of the fluorescence intensity of NG2 with Image J software. Data are presented as the mean ± SEM. *n* = 3 per group. * *p* < 0.05 vs. control. # *p* < 0.05 vs. TGFβ1 group.

To further evaluate the involvement of NG2 in TGFβ1-induced BMSC differentiation to MF, *Cspg4* was knocked down by employing siRNA technology. First, we verified the transfected efficiency and confirmed that *Cspg4* siRNA markedly down-regulated its mRNA (Figure 5A) and protein (Figure 5B) expression in TGFβ1-stimulated BMSC. Moreover, TGFβ1-induced *Acta2* and *Col3a1* mRNA expression were significantly reduced in *Cspg4*-knocked down cells, whereas scramble siRNA had no effect on these mRNA expressions (Figure 5A). The protein expression of αSMA was also decreased after the transfection of *Cspg4* siRNA in TGFβ1-treated cells (Figure 5C). Interestingly, *Cspg4* siRNA down-regulated NG2 expression on the cell membrane, whereas there were more positive-stained spots of NG2 in the cytoplasm of TGFβ1-treated BMSC by immunofluorescent staining (Figure 5D). The relative fluorescence intensity of NG2 analyzed by Image-J software (version 1.0) was shown to decrease after the transfection of *Cspg4* siRNA in TGFβ1-treated BMSC (Figure 5E). These results further supported the key role of NG2 in BMSC differentiation to MF brought about by TGFβ1 and strongly suggested that NG2 contributed to TGFβ1-induced differentiation of BMSC to MF in vitro.

### 2.6. The Participation of NG2 in Liver Fibrosis In Vivo

**Figure 6:**
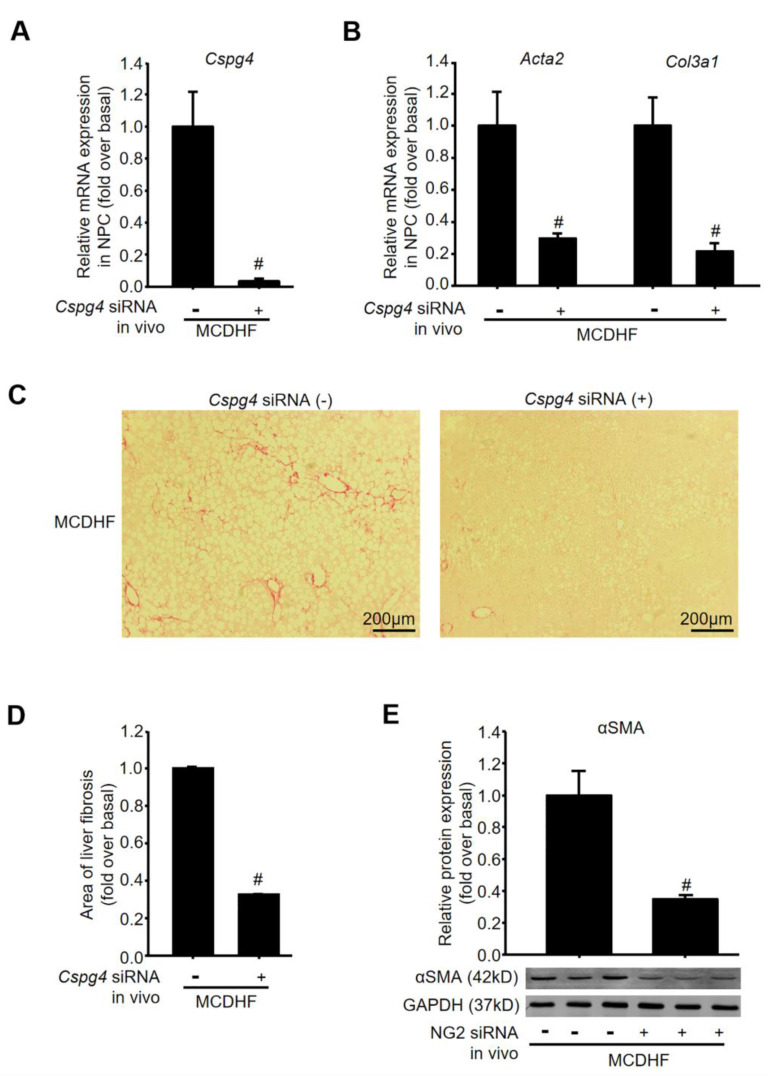
The participation of NG2 in liver fibrosis in vivo. Chemically modified and stable *Cspg4* siRNA (1 μg/g BW) was injected into the mouse tail vein before feeding an MCDHF diet. (**A**) *Cspg4* mRNA levels were measured by qRT-PCR with or without the injection of *Cspg4* siRNA in liver nonparenchymal cells of MCDHF mice. (**B**) The mRNA expression of fibrotic parameters, including *Acta2* and *Col3a1* with or without the injection of *Cspg4* siRNA in nonparenchymal cells of MCDHF mice. (**C**) Representative images of Sirius Red staining in the absence or presence of *Cspg4* siRNA in MCDHF mice. (**D**) Quantitative analysis of fibrotic area with Image-Pro Plus software in Sirius Red-stained liver sections. (**E**) The protein expression of αSMA with or without the injection of *Cspg4* siRNA in MCDHF mice. Data are presented as the mean ± SEM. *n* = 4 per group. # *p* < 0.05 vs. MCDHF group.

**Figure 7:**
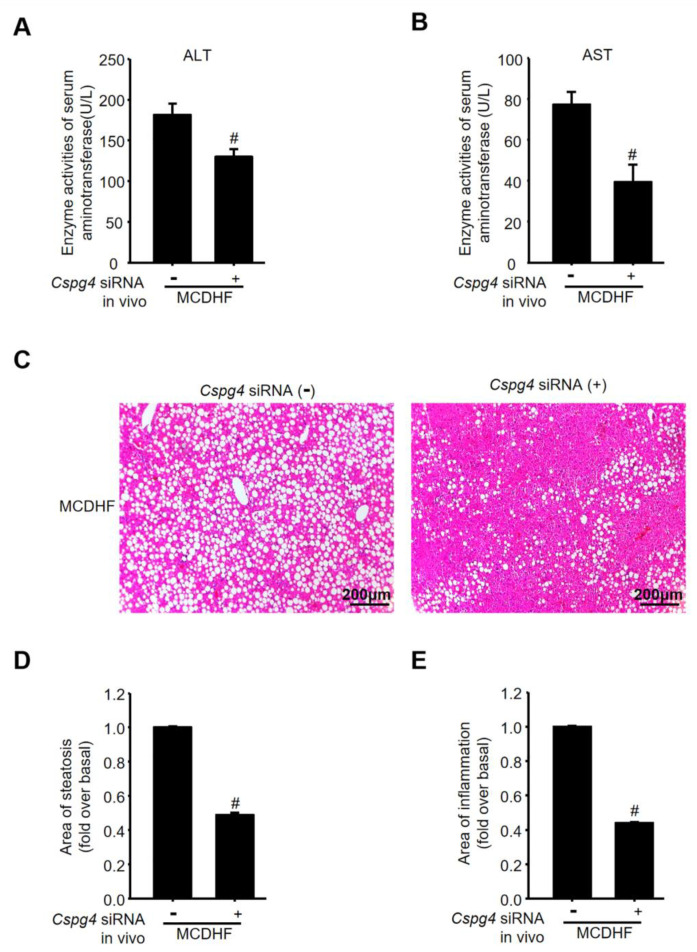
The participation of NG2 in liver fibrosis in vivo. Chemically modified and stable *Cspg4* siRNA (1 μg/g BW) was injected into the mouse tail vein before feeding an MCDHF diet. (**A**) Serum ALT activity in MCDHF mice with or without the injection of *Cspg4* siRNA. (**B**) Serum AST activity in MCDHF mice with or without the injection of *Cspg4* siRNA. (**C**) Representative H&E-stained liver sections in the absence or presence of *Cspg4* siRNA in MCDHF mice. Quantitative analysis of liver steatosis (**D**) and inflammation (**E**) with Image-Pro Plus software in H&E-stained liver sections. Data are presented as the mean ± SEM. *n* = 4 per group. # *p* < 0.05 vs. MCDHF group.

Finally, we verified the effects of NG2 on the underlying liver fibrosis process in vivo, through the injection of chemically modified and stable *Cspg4* siRNA into MCDHF mice, since fatty liver injury has become a common cause of chronic liver disease globally [26,27]. Liver nonparenchymal cells were isolated according to the procedure described in Materials and Methods. The mRNA expression of *Cspg4* in the nonparenchymal cells was examined by qRT-PCR and decreased to 3.4% of normal levels after the injection of *Cspg4* siRNA, verifying the effectiveness and specificity of *Cspg4* siRNA in vivo (Figure 6A). Moreover, the mRNA expression of *Acta2* and *Col3a1* in nonparenchymal cells was explored by qRT-PCR, showing that the mRNA levels of MF marker and fibrotic parameters were markedly attenuated after the injection of *Cspg4* siRNA in MCDHF mice (Figure 6B). Hepatic collagen deposition was evaluated by morphometric analysis with Sirius Red staining and was quantified by digital image analysis, showing that collagen deposition was markedly attenuated after administration of *Cspg4* siRNA in MCDHF mice (Figure 6C,D). The protein expression of αSMA was also dramatically reduced after the administration of *Cspg4* siRNA (Figure 6E). αSMA is a typical marker of MF, thus the reduction in its expression represents the reduction in the number of MF. Consistently, the biochemical parameters indicative of liver injury, including alanine aminotransferase (ALT, Figure 7A) and aspartate aminotransferase (AST, Figure 7B) were decreased after the administration of *Cspg4* siRNA in MCDHF mice. H&E-stained sections (Figure 7C) showed a significant decrease in liver steatosis (Figure 7D) and inflammation (Figure 7E) following the administration of *Cspg4* siRNA in MCDHF mice. Altogether, these data indicated a critical role for NG2 in chronic liver injury, showing that inhibition of NG2 attenuated liver fibrosis in MCDHF mice.

## 3. Discussion

The present study characterizes NG2 expression and function in human and mouse fibrotic liver and establishes NG2 as a powerful regulator for BMSC differentiation to MF in MCDHF mouse models of liver fibrosis. Importantly, inhibition of NG2 by its specific siRNA alleviates liver fibrosis in MCDHF mice, as demonstrated by the decreased expression of fibrotic parameters, collagen deposition, serum transaminase levels, liver steatosis and inflammation after the administration of *Cspg4* siRNA in MCDHF mice.

The origin of MF in liver fibrosis is presumed to be from local activation of residential hepatic stellate cells, fibroblasts, epithelial-mesenchymal transition, or derived from bone marrow cells [28]. Our previous studies have proved that BMSC is a source of MF in the fibrotic liver, thus leading to the exacerbation of liver disease [6,7,8]. Here we identify the expression of NG2 in BMSC-derived MF and characterize the positive regulation of NG2 on BMSC differentiation to MF during liver fibrosis. The mRNA expression of *CSPG4* and fibrosis markers including *ACTA2*, *COL3A1* and *TGFB1* is positively correlated in both human and mouse injured liver. Human liver samples are obtained from patients with chronic liver disease of different etiologies, including chronic HBV, HCV infections, alcoholic, cryptogenic, cholestatic, drug-induced and autoimmune liver disease. Mouse liver injury is induced by feeding an MCDHF diet, CCl_4_ injection or BDL operation, which have all been proven to be typical liver injury models [29]. Thus, our results show the universal regulation of NG2 on BMSC differentiation to MF regardless of species and the etiology of liver diseases, suggesting the great potential of NG2-based therapies for liver diseases. In line with our results, NG2 is also reported as an independent and powerful prognostic marker for disease progression and overall survival for patients with primary HCC [30], which further highlights the significance of NG2 in liver disease. Most importantly, the data collected and described in this study has a huge potential for the development of computational models. Specifically, patients could be classified according to the expression level of NG2 and predict the therapeutic effects and adverse reactions among different patient classifications, laying a foundation for evaluating whether NG2-targeted therapy can be applied clinically.

Regarding the regulatory mechanism of NG2 in BMSC differentiation to MF, here we observed the decreased expression of NG2 on the cell membrane of TGFβ1-treated BMSC after the transfection of *Cspg4* siRNA. At the same time, we unexpectedly found more positive-stained spots of NG2 in the cytoplasm of TGFβ1-treated BMSC after the transfection of *Cspg4* siRNA. This membrane-to-cytoplasm trans-location of NG2 was first documented in the present study and might be one of the reasons accounting for the regulatory mechanism of NG2 in BMSC differentiation to MF. A similar mechanism (in the opposite direction) is observed in thyroid cancer cells, where the intracellular retention of Na^+^/I^+^ symporter (NIS) was observed in the de-differentiation of cells, but in the normal cells, NIS is retained in the plasma membrane [31]. Further studies would be needed to explore this issue.

NG2 is regarded as one of the most widely used molecular markers expressed in pericytes, which surround blood vessels, embed within the basement membrane of vasculature and are adjacent to endothelial cells [32,33]. Pericytes have recently come into focus as regulators of vascular morphogenesis and function during various development, homeostasis and disease processes [34,35]. In the present study, these NG2-positive cells in the fibrotic liver of MCDHF mice might be a part of pericytes and play a critical role in liver fibrosis-associated angiogenesis. Thus, further investigation will be needed to characterize these NG2-positive cells and their role in liver angiogenesis in our mouse models. Moreover, PDGFRβ is also widely used to define pericytes [36]. Our previous study has shown that a large proportion of PDGFRβ-positive cells in the injured liver of MCDHF mice are of BMSC origin, and these PDGFRβ-positive cells contribute to liver angiogenesis and fibrosis [25]. However, whether these NG2-positive cells and PDGFRβ-positive cells in the injured liver represent the same population is still unclear and needs to be further studied. Further experiments will be considered to decipher these NG2-expressing cells by single-cell sequencing technology.

In conclusion, we reveal the essential role of NG2 in BMSC differentiation to MF during liver fibrogenesis. Inhibition of *Cspg4* suppresses BMSC differentiation and attenuates liver fibrosis in vitro and in vivo, which indicates NG2 as a target for the treatment of liver disease.

## 4. Materials and Methods

### 4.1. Mouse Models

Liver fibrosis models were induced by an MCDHF diet (*n* = 3 per group), CCl_4_ administration (*n* = 4 for CCl_4_ 0 day, 5 for CCl_4_ 3 days, 6 for CCl_4_ 1, 2, 7, 14 days and 28 days control per group, 9 for 28 days) or BDL operation (*n* = 3 for BDL 0, 3 days per group, 4 for BDL 1, 14 days per group, 5 for BDL 7 days). In detail, mice were fed either a control diet or an MCDHF diet (A06071309, Research Diet Inc., New Brunswick, NJ, USA) containing 46 kcal% fat, 18 kcal% protein and 36 kcal% carbohydrates. To induce the CCl_4_-induced liver injury model, mice received intraperitoneal injections of 1 μL per gram body weight (BW) of a CCl_4_/olive oil (OO) mixture (1:9 *v/v*) twice per week. For BDL models, mice were anesthetized to receive a midline laparotomy, and then the common bile duct was exposed and ligated three times. Two ligatures were placed in the proximal portion of the bile duct, and one ligature was placed in the distal portion of the bile duct. The bile duct was then cut between the ligatures. The abdomen was closed in layers, and mice were allowed to recover on a heating pad. Mice were anesthetized to collect blood and liver samples at indicated time points. Mouse chemically modified and stable siRNAs of *Cspg4* and Invivofectamine 3.0 reagent were from Invitrogen (Thermo Fisher Scientific, Waltham, MA, USA). The solution of siRNA duplex (1.2 mg/mL) 50 μL was mixed with 50 μL Buffer. The solution was mixed with 100 μL Invivofectamine 3.0 reagent, then incubated at 50 °C for 30 min. The complex was diluted with 1 mL PBS. The solution (1 μg/g BW) was injected into the mouse tail vein before feeding the MCDHF diet.

Another group of mice received lethal irradiation (8Gy), and then immediately received transplantation by a tail vein injection of cell mixture (1 × 10^6^ BMSC from EGFP transgenic mice and 1.10 × 10^7^ whole bone marrow cells from wild-type mice, in which BMSC had been removed). Anti-CD146 microbeads (Miltenyi Biotec, Bergisch Gladbach, Germany) were used to purify BMSC or remove BMSC from whole bone marrow cells by immunomagnetic cell sorting. Four weeks later, the bone marrow was reconstructed, and the chimera mice with EGFP-labeled BMSC were subjected to liver injury. All animals received human care and all animal study protocols were approved by the ethics committee of Capital Medical University (approval number: AEEI-2020-149).

### 4.2. Human Liver Specimens

Human fibrotic samples (fibrosis stage: F2-4) were obtained from the livers of 22 patients undergoing liver biopsy (11 men, 11 women; mean age, 58 years; range, 29–72 years). Detailed patient information is given in Supplementary Table S1. Fibrosis was consecutive to chronic HBV, HCV, alcoholic, cryptogenic, cholestatic, drug-induced and autoimmune liver disease. Normal liver samples were collected from six patients undergoing hepatic resection for hepatic hemangioma. All subjects gave their informed consent for inclusion before they participated in the study. Written informed consent has been obtained from the patient(s) to publish this paper. The study was conducted in accordance with the Declaration of Helsinki, and the protocol was approved by the Ethics Committee of Beijing Shijitan Hospital, Capital Medical University, Beijing, China (project identification code: 2018EC-1).

### 4.3. BMSC Isolation and Culture

ICR mice were anesthetized, and whole BM cells were extracted from the tibias and femurs by using a 25G needle to flush with culture medium. The cells were filtered through a 70 μm nylon mesh and washed with PBS containing 2% FBS. Then BMSC were cultured and used for experiments from passages 3 to 6. Characterization of BMSC was performed by flow cytometry analysis as previously described [7].

### 4.4. qRT-PCR

Total RNA from liver tissue, BMSC and liver non-parenchymal cells, which were separated using low-speed centrifugation and 40% percoll density gradient centrifugation, was extracted, then qRT-PCR was performed. Primers were as follows: 18S rRNA: sense, 5′-GTA ACC CGT TGA ACC CCA TT-3′; antisense, 5′-CCA TCC AAT CGG TAG TAG CG-3′. Mouse *Cspg4*: sense, 5′-GGG CTG TGC TGT CTG TTG A-3′; antisense, 5′-TGA TTC CCT TCA GGT AAG GCA-3′. Mouse *Acta2*: sense, 5′-ATG CTC CCA GGG CTG TTT T-3′; anti-sense, 5′-TTC CAA CCA TTA CTC CCT GAT GT-3′. Mouse *Col3a1*: sense, 5′-TGA AAC CCC AGC AAA ACA AAA-3′; antisense, 5′-TCA CTT GCA CTG GTT GAT AAG ATT AA-3′. Mouse *Tgfb1*: sense, 5′-TGC GCT TGC AGA GAT TAA AA-3′; antisense, 5′-TCA CTG GAG TTG TAC GGC AG-3′. Human *CSPG4*: sense, 5′-CTT TGA CCC TGA CTA TGT TGG C-3′; antisense, 5′-TGC AGG CGT CCA GAG TAG A-3′. Human *TGFB1*: sense, 5′-AGT TCA AGC AGA GTA CAC ACA GCA T-3′; antisense, 5′-AGA GCA ACA CGG GTT CAG GTA-3′. Human *ACTA2*: sense, 5′-CTG GCA TCG TGC TGG ACT CT-3′; antisense, 5′-GAT CTC GGC CAG CCA GAT C-3′. Human *COL3A1*: sense, 5′-GGG AAT GGA GCA AAA CAG TCT T-3′; antisense, 5′-CCA ACG TCC ACA CCA AAT TCT-3′.

### 4.5. Immunofluorescence Staining

Liver samples were fixed in 4% paraformaldehyde and embedded in Tissue Tek OCT compound. Five micrometers of frozen section were used for immunofluorescence. The liver sections or cultured BMSC were blocked with 2% BSA and then incubated with anti-NG2 polyclonal antibody (1:100, Millipore, Billerica, MA, USA) and FITC-conjugated AffiniPure donkey anti-rabbit IgG antibody (1:100; Jackson Immunoresearch, West Grove, PA, USA) as a secondary antibody. Immunofluorescent detection of αSMA was performed using a Vector M.O.M. (mouse-on-mouse) immunodetection kit (Vector Laboratories, Burlingame, CA, USA) and a 1:200 dilution of a monoclonal antibody to αSMA (Sigma, St. Louis, MO, USA). The samples were covered with Vectashield mounting medium containing DAPI and observed under a confocal microscope (TCS SP5, Leica, Wetzlar, Germany). The proportion of NG2^+^αSMA^+^ cells accounting for total NG2^+^ cells and the proportion of NG2^+^EGFP^+^ cells accounting for total EGFP^+^ cells were measured by Image-Pro Plus software. The relative fluorescence intensity of NG2 was measured by Image-J software.

### 4.6. Western Blot Analysis

Western blot analysis of NG2 was performed with 200 μg of protein extract using polyclonal antibodies to NG2 (1:500, Abcam, Cambridge, UK), αSMA (1:1000, Proteintech, Rosemount, IL, USA) and the appropriate IRDyeTM 800-conjugated secondary antibody (1:10,000). Signals were detected using the Odyssey Imaging System (LI-COR Biosciences, Lincoln, NE, USA) and analyzed with Odyssey software. Results were normalized relative to glyceraldehyde-3-phosphate dehydrogenase (GAPDH) (mouse anti-GAPDH monoclonal antibody, 1:1000; Sigma, St. Louis, MO, USA) or β-tubulin (mouse anti-β-tubulin monoclonal antibody, 1:1000; TransGen Biotech, Beijing, China) expression to correct for variations in protein loading and transfer.

### 4.7. Quantitative Analysis of Liver Fibrosis, Steatosis and Inflammation

Liver tissues were fixed in PBS containing 4% paraformaldehyde for 24 h and embedded in paraffin. Liver sections (5 μm) were stained with Sirius red for collagen visualization, and H&E for steatosis and inflammation. The steatosis, fibrotic and inflammatory areas were assessed by computer-assisted image analysis with Image-Pro Plus software. The mean value of randomly selected areas per sample was used as the expressed percentage of steatosis, fibrotic, or inflammatory area.

### 4.8. ALT and AST Measurement

ALT and AST levels were detected by BS-200 Chemistry Analyzer (Mindray, Shenzhen, China).

### 4.9. Statistical Analysis

The results were expressed as mean ± standard error of the mean (SEM). Statistical analyses were carried out using Statistical Package for Social Sciences (SPSS, version 25) software. A two-sided Student’s *t*-test was used to analyze differences between two groups, and a one-way analysis of variance (ANOVA) followed by a post hoc LSD test was used when more than two groups were compared. Correlation coefficients were calculated by the Pearson test. *p* < 0.05 was considered to be significant. All results were verified in at least three independent experiments.

## Data Availability

Not applicable.

## Supplementary Materials

The following supporting information can be downloaded at: https://www.mdpi.com/article/10.3390/ijms24021177/s1.
