# Supplementary material for: Neuron-Glial Antigen 2 Participates in Liver Fibrosis via Regulating the Differentiation of Bone Marrow Mesenchymal Stem Cell to Myofibroblast
Source: Int J Mol Sci. 2023 Jan 7;24(2):1177. doi: 10.3390/ijms24021177 (PMC9864665; doi:10.3390/ijms24021177)
Supplement: Supplementary file 1 [file ijms-24-01177-s001.zip › ijms-2037684-supplementary.pdf]

Supplementary Table S1. The patients' information

	Sex	Age	Etiology of the disease	Stage
1	male	48	HBV	F2F3
2	female	72	autoimmune	F2
3	male	58	HBV	F4
4	male	51	HBV	F3F4
5	female	58	HCV	F2F3
6	female	50	HBV	F2F3
7	female	70	HBV	F3F4
8	female	59	autoimmune	F3F4
9	male	29	HBV	F3F4
10	male	52	cholestatic	F2F3
11	male	70	drug-induced	F2F3
12	female	62	autoimmune	F2F3
13	female	64	HCV	F2F3
14	female	68	cryptogenic	F3F4
15	male	42	HBV	F2F3
16	male	57	alcoholic	F2F3
17	female	67	cryptogenic	F2F3
18	male	51	HCV	F2
19	female	48	cryptogenic	F3F4
20	male	62	HCV	F2F3
21	male	68	HBV	F4
22	female	68	cryptogenic	F3
23	female	49	hepatic hemangioma	
24	female	41	hepatic hemangioma	
25	male	29	hepatic hemangioma	
26	female	59	hepatic hemangioma	
27	male	49	hepatic hemangioma	
28	male	54	hepatic hemangioma	

The patients' information includes sex, age, etiology of the disease and stage. Human fibrotic samples (fibrosis stage: F2-4) were obtained from livers of 22 patients undergoing liver biopsy (11 men, 11 women; mean age, 58 years; range, 29-72 years). Fibrosis was consecutive to chronic HBV (n=8), HCV (n=4), alcoholic (n=1), cryptogenic (n=4), cholestatic (n=1), drug-induced (n=1), and autoimmune (n=3) liver disease. Normal liver samples were collected from 6 patients undergoing hepatic resection for hepatic hemangioma. All subjects gave their informed consent for inclusion before they participated in the study. Written informed consent has been obtained from the patient(s) to publish this paper The study was conducted in accordance with the Declaration of Helsinki, and the protocol was approved by the Ethics Committee of Beijing Shijitan Hospital, Capital Medical University, Beijing, China (project identification code: 2018EC-1).
